# Adverse Childhood Experiences and Their Effect on Irritable Bowel Syndrome Among Saudi Arabian Adults

**DOI:** 10.7759/cureus.25791

**Published:** 2022-06-09

**Authors:** Mohammed A Alsubaie, Hussain A Alkhalifah, Abdulah H Ali, Mohammed A Bahabri, Badr A Alharbi, Sulhi A Alfakeh

**Affiliations:** 1 Faculty of Medicine, King Abdulaziz University, Jeddah, SAU; 2 Psychiatry, King Abdulaziz University, Jeddah, SAU

**Keywords:** saudi arabia, ibs, irritable bowel syndrome, aces, adverse childhood experiences, neglect, child abuse

## Abstract

Background

Adverse childhood experiences (ACEs) are traumatic events that occur before 18 years of age. ACEs have been associated with many negative health problems, including the development of chronic diseases, such as irritable bowel syndrome (IBS), a functional gastrointestinal disorder characterized by abdominal pain. We investigated the prevalence of ACEs among patients with IBS, identified the types of ACEs commonly related to patients with IBS, and further assessed the impact of ACEs on IBS severity.

Methodology

A cross-sectional study was performed. The study targeted patients with IBS aged ≥ 18 years who were recruited from gastroenterology outpatient clinics at King Abdulaziz University Hospital. Adults were contacted and invited to take part in the study by completing a survey. Data were collected using two validated questionnaires, the ACE questionnaire for adults and the IBS symptom severity scoring system.

Results

The study included 109 patients with IBS (59.6% females). The prevalence of ACEs (patients with IBS exposed to at least one ACE) was 63.3%. The most prevalent type was emotional abuse (34.9%), followed by both physical abuse and emotional neglect (28.4%). Females reported significantly more ACEs (p = 0.035) than males. The overall IBS symptoms (r = 0.195, p = 0.043) and abdominal pain (r = 0.240, p = 0.012) severity were significantly correlated with total ACEs score.

Conclusions

Our findings point to a probable association between ACEs exposure and IBS, demonstrating their long-term impacts on symptoms severity. Further studies are needed to acquire a better understanding of the potential impact of ACEs on IBS.

## Introduction

Adverse childhood experiences (ACEs) refer to stressful or traumatic events that occur during childhood before 18 years of age [1]. ACEs were first defined by the 1998 Kaiser Permanente and the Centers for Disease Control and Prevention major ACE study, and the results increased interest in the consequences of ACEs on health outcomes [2]. ACEs were then classified into three categories of abuse (emotional, physical, and sexual abuse), five household challenges (household substance abuse, family violence, mentally ill household members, criminal household member, and parental separation or divorce), and two types of neglect (emotional and physical neglect) [3]. According to a national study, 80% of Saudi adults have been exposed to at least one ACE, with 29% having been exposed to four or more ACEs [4]. Furthermore, Saudi adults who had experienced four or more ACEs were at higher risk of developing diabetes, hypertension, and chronic respiratory diseases [5]. There is considerable evidence that toxic stress or frequent traumatic events can irreversibly damage the growing brain and alter the functionality of the immune, neurological, and endocrine systems. Thus, individuals who live in a stressful or toxic atmosphere will be exposed to a higher risk of chronic diseases and premature death [3]. Besides the effect on a person’s health, the lifelong expenses associated with ACEs are significant. Indeed, the lifetime expenses of a person abused as a child are estimated to be approximately $210,012 [6].

Irritable bowel syndrome (IBS) is one of the most common gastrointestinal functional disorders [7]. It is characterized by chronic, recurrent abdominal discomfort, pain, and distorted bowel habits in the absence of other organic gastrointestinal diseases [8]. IBS is generally categorized into four subtypes based on the individual’s dominant stool pattern: IBS with constipation (IBS-C), diarrhea (IBS-D), mixed bowel habit (IBS-M), and unclassified IBS (IBS-U) [9]. The etiology of IBS is unclear; however, it is assumed that the biopsychosocial model affects its development [10]. Thus, previous environmental events and psychosocial factors are considered to be crucial in its development.

A relationship between ACEs and IBS has been established throughout the years, showing an association between the two. Previous studies have found that patients with IBS have a greater prevalence of ACEs with respect to emotional, physical, and sexual abuse than the general community [11-13]. In a recent Iranian study, emotional abuse and emotional neglect were found to be the most common ACEs encountered in patients with IBS [14].

Although some studies conducted in Saudi Arabia have estimated the prevalence of ACEs and their relationship with other chronic diseases, no previous studies have linked their relationship with IBS, despite their proven association. Therefore, this study aims to 1) estimate the prevalence of ACEs among patients with IBS, 2) identify the types of ACEs most commonly associated with IBS patients, 3) compare the gender differences in the association of ACEs in patients with IBS, and 4) investigate the impact of ACEs on the severity of IBS symptoms.

## Materials and methods

Study design, setting, and population

This cross-sectional study was performed between June and July 2021. Altogether, 308 patients were contacted, of which 109 agreed to participate and completed the survey. The study targeted male and female patients with IBS aged ≥ 18 years who were recruited from gastroenterology outpatient clinics at King Abdulaziz University Hospital (KAUH) in Jeddah, Saudi Arabia. However, we excluded patients aged > 65 years, those with severe cognitive impairment, or those with other chronic gastrointestinal diseases.

Data collection method

All patient-related information (age, gender, nationality, date of admission, body mass index (BMI), diagnosis of IBS, and phone numbers) was obtained from the KAUH database. Adults were contacted and invited to take part in the study by completing a self-administered survey. First, oral consent from every patient was taken through a phone call after explaining the aims of the study. After that, an anonymous survey using Google forms was sent to them through SMS messages. No incentives were offered to the participants to take part in the study. The survey was anonymous, and the responses were treated confidentially. The survey was divided into three sections: the first section enquired about personal data (age and gender); the second evaluated ACEs exposure before 18 years of age using the ACE questionnaire for adults [15]; the third measured IBS symptom severity using the IBS Severity Scoring System (IBS-SSS) questionnaire (Appendices) [16].

Questionnaires

ACE Questionnaire for Adults

This questionnaire was used to assess the number of ACEs exposure before 18 years. The ACE questionnaire was adapted from Kaiser Permanente and the Centers for Disease Control and Prevention [2]. We used the Arabic version of the questionnaire compiled by The Office of the California Surgeon General and the Department of Health Care Services, in collaboration with the ACEs Aware Clinical Advisory Subcommittee. The questionnaire is available on their website [15].

The questionnaire consists of 10 questions covering the 10 categories of ACEs (childhood emotional, physical, and sexual abuse, emotional and physical neglect, household substance abuse, mentally ill household members, criminal household members, family violence, and parental separation or divorce). The ACE score is computed by assigning one point to each category (“yes” = 1 or “no” = 0). The 10 indicators are then summed to produce a total ACE score (ranging from 0 to 10). ACEs can therefore be labeled as either present (total ACE score ≥ 1) or absent (total ACE score = 0).

IBS Severity Scoring System (IBS-SSS)

This is a valid and reliable tool for evaluating the severity of IBS gastrointestinal symptoms. It includes five items that measure the severity of abdominal pain, frequency of abdominal pain, severity of abdominal distension, dissatisfaction with bowel habits, and interference with quality of life. Each question is assessed on a scale from 0 to 100, with a total score ranging from 0 to 500. The higher the total score, the more severe the symptoms. Patients are then classified according to their total score into mild (75-175), moderate (176-300), or severe (above 300). Patients scoring less than 75 can be considered to be in remission [16]. The questionnaire was translated into Arabic, which was then back-translated into English by two independent translators.

Statistical analysis

Microsoft Excel 2016 (Microsoft Corporation, Redmond, WA) was used for data entry and IBM SPSS Statistics version 21 (IBM Corp., Armonk, NY) for statistical analysis.

The descriptive statistics of the sociodemographic and clinical characteristic variables, including gender, age, diagnosis of IBS, and nationality, were calculated to determine frequency and percentages. The descriptive statistics of ACEs prevalence (10 ACEs categories) were also calculated to determine the frequency and percentages. For the continuous variables, measures of central tendency were computed. Normal distribution was assessed using histograms and the Shapiro-Wilk test.

Bivariate analysis was conducted using the chi-square test to determine the gender differences in each ACE category. Moreover, an independent sample t-test was performed to determine the gender differences in the total ACE scores and to evaluate the impact of each ACE category on the severity of IBS symptoms. Additionally, Pearson’s correlation coefficient was used to evaluate the relationship between total ACEs and IBS-SSS scores and was considered statistically significant at p < 0.05.

Ethical approval

The study was approved by the Faculty of Medicine Research Ethics Committee, King Abdulaziz University, Jeddah, Saudi Arabia (Reference number 638-20). Verbal consent was first obtained through a phone call. Then the participants agreed to participate one more time by filling out the survey and giving their approval.

## Results

Participant characteristics

This study included 65 women (59.6%). The mean age of the patients with IBS was 37 years ± 10.89. Most of the participants were Saudi nationals (81.7%), and 57.8% were diagnosed with IBS-D. Table 1 outlines the participants’ sociodemographic and clinical characteristics.

**Table 1:** Participants’ sociodemographic and clinical characteristics Abbreviations: N: number, SD: standard deviation, BMI: body mass index

Characteristics	N	%
Gender		
Male	44	40.4
Female	65	59.6
Age (years), Mean±SD	37.1 ± 10.89	
18-29	30	27.5
30-44	54	49.5
45-65	25	22.9
Nationality		
Saudi	89	81.7
Non-Saudi	20	18.3
Diagnosis description		
Irritable bowel syndrome with diarrhea	63	57.8
Irritable bowel syndrome without diarrhea	46	42.2
BMI, Mean±SD	27.79 ± 7.55	

Adverse childhood experiences

The mean ACE score among the participants was 1.77 ± 1.95, and the prevalence of ACEs (patients with IBS exposed to at least one ACE) was 63.3%. Females outnumbered males in this study (70.8% vs. 52.2%). Moreover, 15.6%, 21.1%, 9.2%, and 17.4% of the patients with IBS had ACEs scores of 1, 2, 3, and ≥ 4, respectively. Males outnumbered females in having an ACE score of 0 (47.7% vs. 29.2%). When the total ACEs scores were compared between males and females, female patients with IBS reported significantly higher ACEs scores than male patients with IBS (p = 0.035).

**Figure 1:**
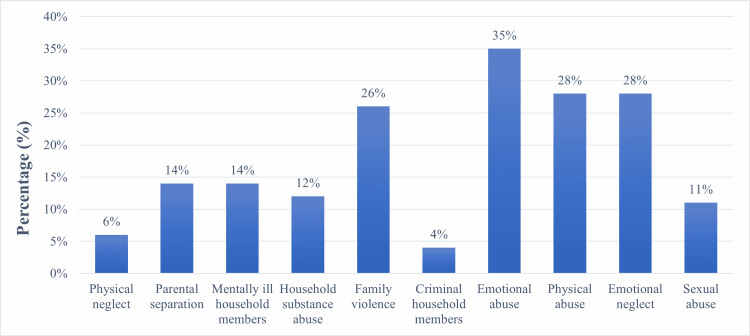
Prevalence of ACEs among IBS patients. Abbreviations: ACEs: adverse childhood experiences, IBS: irritable bowel syndrome

Figure 1 shows the prevalence of ACEs among patients with IBS, with emotional abuse being the most common (34.9%), followed by physical abuse (28.4%) and emotional neglect (28.4%).

**Figure 2:**
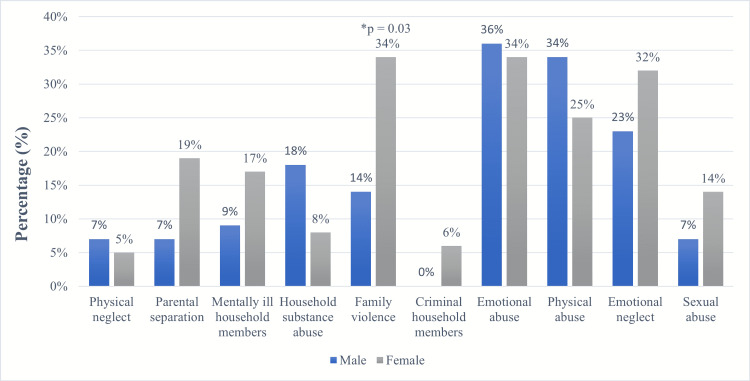
Prevalence of different ACEs among IBS patients separated by gender Abbreviations: ACEs: adverse childhood experiences, IBS: irritable bowel syndrome *A p-value less than 0.05 is considered statistically significant

We further stratified all ACEs for additional exploratory analyses by comparing each type of frequency by gender, as shown in Figure 2. Only a few differences were found between the 10 types of ACEs; females reported significantly higher family violence compared to males (33.8% vs. 13.6%, p = 0.03). Also, female participants reported higher rates of parental separation (18.5% vs. 6.8%, p = 0.15), emotional neglect (32.3% vs. 22.7%, p = 0.38), and household mental illness (16.9% vs. 9.1%, p = 0.38) than male participants. However, these differences were not statistically significant.

Irritable bowel syndrome severity

The total mean (IBS-SSS) score for both genders was 241.47 ± 130.241, with a higher mean of the total score (258.31 ± 134.043) for females than for males (216.59 ± 121.654). Based on their symptom intensity, patients were then categorized into four categories: in remission, 11 (10.1%); mild, 27 (24.8%); moderate, 36 (33%); and severe, 35 (32.1%).

Association of ACEs and IBS symptoms severity

**Figure 3:**
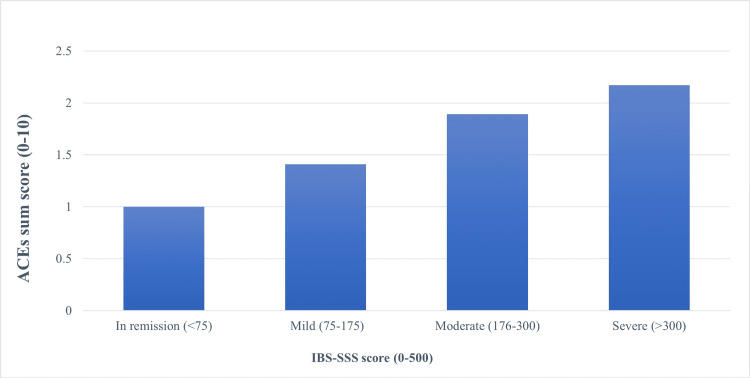
Association of ACEs and IBS-SSS scores in IBS patients. Abbreviations: ACEs: adverse childhood experiences, IBS: irritable bowel syndrome severity scoring system

Pearson correlation coefficient demonstrated weak but significant correlations between the total ACEs score and the severity of overall IBS symptoms (r = 0.195, p = 0.043) and abdominal pain (r = 0.240, p = 0.012). The mean ACEs scores for each IBS category were as follows: in remission, 1.00 ± 1.18; mild, 1.41 ± 1.90; moderate, 1.89 ± 1.87; and severe, 2.17 ± 2.17. As illustrated in Figure 3, the mean ACE score increased as the severity of IBS increased.

We further analyzed each ACE category with the severity of IBS symptoms, as presented in Table 2. Patients with IBS who were emotionally neglected (p = 0.003) or sexually abused (p = 0.011) had more severe IBS symptoms than those who were not.

**Table 2:** Correlation between different types of ACEs with the severity of IBS symptoms Abbreviations: ACEs: adverse childhood experiences, IBS: irritable bowel syndrome severity scoring system, N: number, SD: standard deviation *A p-value less than 0.05 is considered statistically significant

Types of ACEs	N (%)	IBS-SSS score Mean (SD)	p-value
Emotional abuse			
Yes	38 (34.9%)	249.47 (136.875)	0.641
No	71 (65.1%)	237.18 (127.337)	
Physical abuse			
Yes	31 (28.4%)	248.39 (137.552)	0.728
No	78 (71.6%)	238.72 (128.036)	
Sexual abuse			
Yes	12 (11%)	330.83 (135.543)	0.011*
No	97 (89%)	230.41 (125.921)	
Emotional neglect			
Yes	31 (28.4%)	300.00 (115.672)	0.003*
No	78 (71.6%)	218.21 (129.040)	
Physical neglect			
Yes	6 (5.5%)	336.67 (109.301)	0.065
No	103 (94.5%)	235.92 (129.656)	
Parental separation			
Yes	15 (13.8%)	235.33 (138.969)	0.845
No	94 (86.2%)	242.45 (129.554)	
Family violence			
Yes	28 (25.7%)	280.71 (113.266)	0.064
No	81 (74.3%)	227.90 (133.583)	
Household substance abuse			
Yes	13 (11.9%)	228.46 (136.617)	0.703
No	96 (88.1%)	243.23 (130.000)	
Household mental illness			
Yes	15 (13.8%)	266.00 (82.877)	0.276
No	94 (86.2%)	237.55 ( 136.206)	
Criminal household members			
Yes	4 (3.7%)	277.50 (45.735)	0.211
No	105 (96.3%)	240.10 (132.298)	

## Discussion

Our findings revealed that patients with a higher ACEs score experienced more severe IBS symptoms, as the total ACEs score was positively correlated with the overall IBS symptoms' severity and abdominal pain, which is consistent with the findings of similar previous studies [13,17]. This could be due to the increased stress response in patients with IBS having a history of ACEs (i.e., hypothalamic-pituitary-adrenal axis dysregulation) resulting from early-life ACEs exposure [18], which contributes to stress-induced visceral hyperalgesia and intestinal mucosal barrier dysfunction [19].

In this study, emotional neglect and sexual abuse were significantly associated with severe IBS symptoms. The observed relationship between these types of ACEs and IBS severity can be related to their long-lasting effect because events such as sexual abuse could stick in the memory for life, affecting people’s social life and mental health by altering their physiological state and increasing stress [20].

We also found that the prevalence of ACEs in patients with IBS was 63.3%. Compared with other studies conducted on patients with IBS, our results demonstrated an almost similar prevalence of ACEs to a German study (63.8%) [21] but it was less prevalent than an American study (75.68%) [13].

Furthermore, females reported significantly more ACEs than male patients with IBS. This coincides with Berens et al.’s study [21]. A possible cause could be that different types of ACEs are related to one gender more than the other. For example, our study results showed that females are more prone to sexual abuse (14% in females vs. 7% in males). This has also been demonstrated in other studies [11,13,21]. Other possible explanations for these results are that females are more susceptible to the effects of ACEs and, therefore, develop psychopathologies [22-23]. Others hypothesize that males and females react to stress in different ways, with females being more inclined to internalize stress symptoms (e.g., depression) and males more willing to externalize stress reactions (e.g., aggressive behavior) [24].

Moreover, this study confirmed that females reported significantly higher family violence (34%) than males (14%). This could be due to the rising financial and social concerns among family members, which could increase the chances of exposure to various forms of abuse [25]. The Middle Eastern culture may explain this relationship, as young females in the Middle East spend more time at home. Therefore, they might witness family violence more than males, who spend most of their time outside.

Out of the 10 ACE categories in our study, emotional abuse was found to be the most common, followed by physical abuse and emotional neglect. This correlates with the findings of an Iranian study [14]. A possible explanation for this result may be that physical and emotional abuse are widely used by parents as disciplinary methods in the Middle East. These actions can have a long-lasting effect on the child even after they grow up, which could alter their mental state and cause symptoms of depression and anxiety. The importance of this point was demonstrated in previous studies in which patients with IBS had greater levels of anxiety and depression symptoms than healthy controls [11,13,21].

Strengths and limitations

This study vividly analyzed the association of IBS with ACEs in the Saudi community, and this study is the first study of its kind from the Saudi region to prove this association convincingly. However, our study has some limitations. First, because of the nature of the cross-sectional study design, no causal inferences could be identified. Second, the questionnaire consisted of retrospective questions of ACEs; the application of this technique could result in recall bias, leading to underreporting or overreporting.

## Conclusions

Our findings point to a probable association between ACEs and IBS, demonstrating their long-term impacts on symptoms severity. This highlights the need to consider the harmful effects of ACEs on the development and severity of IBS. Therefore, it is important to minimize or prevent ACEs by raising awareness of their consequences and establishing children’s educational programs to make them aware of the positive effects of seeking help from others. Moreover, screening for ACEs in patients with IBS should be considered. This will provide the necessary psychological therapy, if needed, to decrease gastrointestinal symptom severity. We recommend a longitudinal prospective cohort study to be conducted for a more reliable understanding of the relationship between ACEs exposure and IBS development.

## Appendices

Research questionnaire

The aim of this study is to measure the prevalence of adverse childhood experiences and their association with irritable bowel syndrome. All Information collected from this survey will be treated with the utmost confidentiality and will only be used for research purposes.

The study was ethically approved by the Scientific Research Ethics Committee at the Faculty of Medicine, King Abdulaziz University.

Section 1: personal data

----------------------------------------------------------------------------------------------------------------

Gender:

1- Male

2- Female

Age (in numbers): ...........

Section 2: adverse childhood experience questionnaire

Below is a list of 10 categories of Adverse Childhood Experiences (ACEs). From the list below, please answer "yes" next to each ACE category that you experienced prior to your 18th birthday.

----------------------------------------------------------------------------------------------------------------

Before the age of 18, Did you feel that you didn’t have enough to eat, had to wear dirty clothes, or had no one to protect or take care of you?

1. Yes

2. No

Before the age of 18, Did you lose a parent through divorce, abandonment, death, or other reason?

1. Yes

2. No

Before the age of 18, Did you live with anyone who was depressed, mentally ill, or attempted suicide?

1. Yes

2. No

Before the age of 18, Did you live with anyone who had a problem with drinking or using drugs, including prescription drugs?

1. Yes

2. No

Before the age of 18, Did your parents or adults in your home ever hit, punch, beat, or threaten to harm each other?

1. Yes

2. No

Before the age of 18, Did you live with anyone who went to jail or prison?

1. Yes

2. No

Before the age of 18, Did a parent or adult in your home ever swear at you, insult you, or put you down?

1. Yes

2. No

Before the age of 18, Did a parent or adult in your home ever hit, beat, kick, or physically hurt you in any way?

1. Yes

2. No

Before the age of 18, Did you feel that no one in your family loved you or thought you were special?

1. Yes

2. No

Before the age of 18, Did you experience unwanted sexual contact (such as fondling or oral/anal/vaginal intercourse/penetration)?

1. Yes

2. No

Section 3: irritable bowel syndrome (IBS) severity score

This questionnaire is aimed to help us record and evaluate the severity of your IBS.

The first question will ask you to write down the number of days you were in pain.

Other questions will ask you to evaluate your IBS symptoms on a scale of 0 to 100.

----------------------------------------------------------------------------------------------------------------

On how many of the last 10 days did you get pain? (For example, if you enter 4 it means that you get pain in 4 out of last 10 days, if you get pain every day enter 10)

.................. number of days with pain

How severe has your abdominal (tummy) pain been over the last ten days?

0          10          20          30          40          50          60          70          80          90          100

no pain           not very severe                     quite severe             severe           very severe

How severe has your abdominal distension (bloating, swollen or tight) been over the last ten days?

0          10          20          30          40          50          60          70          80          90          100

no distension          not very severe          quite severe             severe             very severe

How satisfied have you been with your bowel habit (frequency, ease, etc) over the last ten days?

0          10          20          30          40          50          60          70          80          90          100

very happy                     quite happy                          unhappy                          very unhappy

How much has your IBS been affecting/interfering with your life in general over the last ten days?

0          10          20          30          40          50          60          70          80          90          100

not at all                           not much                            quite a lot                           completely  
